# Diagnostic accuracy of dual-energy CT for bone marrow edema in patients with acute knee injury: a systematic review and meta-analysis

**DOI:** 10.1186/s13018-023-04151-3

**Published:** 2023-11-02

**Authors:** Zhizhuo Li, Xuelong Chen, Hui Fang, Chengxin Li, Lijun Shi, Xiaoyu Fan, Xin Xu, Fuqiang Gao, Wei Sun, Jiang Qing

**Affiliations:** 1 State Key Laboratory of Pharmaceutical Biotechnology, Division of Sports Medicine and Adult Reconstructive Surgery, Department of Orthopedic Surgery, Nanjing Drum Tower Hospital, Affiliated Hospital of Medical School, Nanjing University, Nanjing, Jiangsu China; 2 Department of Orthopedics, Peking University China-Japan Friendship School of Clinical Medicine, 2 Yinghuadong Road, Chaoyang District, Beijing, 100029 China; 3 Beijing Key Laboratory of Immune Inflammatory Disease, China-Japan Friendship Hospital, Peking Union Medical College, 2 Yinghuadong Road, Chaoyang District, Beijing, 100029 China; 4 Department of Radiology, Shanghai Jiao Tong University Affiliated Sixth People’s Hospital, 600 Yishan Road, Shanghai, 200233 China; 5 Department of Orthopedics, The First Affiliated Hospital of Zhengzhou University, Zhengzhou, 450052 Henan China

**Keywords:** Acute knee injury, Bone marrow edema, Dual-energy computed tomography, Meta-analysis

## Abstract

**Background:**

Knee injuries are prevalent, and early diagnosis is crucial for guiding clinical therapy. MRI is the diagnostic gold standard for bone marrow edema (BME) in patients with acute knee injuries, yet there are still limitations. Dual-energy CT, a possible viable replacement, is being explored (DECT).

**Methods:**

We systematically retrieved studies from EMBASE, Scopus, PUBMED, and the Cochrane Library and collected gray literatures. In accordance with the PRISMA-DTA standards, a systematic review was conducted between the study's initiation and July 31, 2021, utilizing an MRI reference standard and at least 10 adult patients with acute knee injuries to evaluate the diagnostic effectiveness of DECT for diagnosing BME. Two reviewers collected the study's details independently. For the meta-analysis, a bivariate mixed-effects regression model was utilized, and subgroup analysis was employed to determine the sources of variability.

**Results:**

The research included nine studies that examined 290 individuals between the ages of 23 and 53 with acute knee injuries who had DECT and MRI. Overall, the sensitivity, specificity, and AUC of the BME were 85% (95% confidence interval [CI]: 77–90%), 96% (95% CI: 93–97%), and 0.97 (95% CI: 0.95–0.98), respectively. To account for the assumed diversity of research, there were no statistically significant differences between the comparison groups in terms of specificity and sensitivity.

**Conclusion:**

DECT is a viable alternative to MRI for individuals with acute knee injuries when MRI is inappropriate or unavailable.

## Introduction

The prevalence of knee injury is high and early diagnosis is significant to guide clinical treatment. Early diagnosis and early treatment can prevent the further development of injury, especially for traumatic bone marrow injury without obvious fracture line [1]. MRI can precisely identify bone marrow edema (BME), a widely recognized indicator of minor bone injury such microfracture and hemorrhage focused in the trabecular bone [2]. Hence, MRI is considered the gold standard for finding concealed fractures in patients with acute knee injury [1]. Unfortunately, MRI examinations are lengthy and restricted by medical equipment such as pacemakers. Also, the patient must remain motionless throughout the process, which might be difficult for elderly or trauma patients [2, 3].

Dual-energy CT (DECT) is examined as a possible alternative to MRI with an enhanced three-material decomposition approach that can eliminate elements having photoelectric effects relevant to BME, such as calcium and iodine [4–6]. The clinical use of DECT for measuring BME in adult patients with an acute knee injury is still the subject of discussion and investigation. Prior to using DECT as a replacement for MRI, its accuracy must be carefully established. Thus, the purpose of this systematic review and meta-analysis was to compile and assess available evidence on the sensitivity of DECT for identifying BME in adult patients with acute knee injury and to establish whether MRI is still required for these individuals.

## Methods

This systematic review and meta-analysis was presented according to the Diagnostic Test Accuracy (PRISMA-DTA) standards [7]. The techniques of evidence seeking and data processing discussed in this article were based on the diagnostic test accuracy method developed by the Cochrane Collaboration [8]. This article needed neither ethical approval nor informed consent, as all data were gathered from published sources. Searching for papers, determining eligibility, obtaining data, and evaluating quality were undertaken separately by two researchers. Disagreements were handled through debate until a consensus was reached.

### Search strategy

Four electronic databases, PubMed, EMBASE, Scopus, and Cochrane Library, were combed for items recorded between the creation of the databases and July 31, 2021. The vocabulary and syntax were tailored precisely to the database. The first search contained variants of title/abstract/keywords and medical topic heading phrases, such as "dual energy" AND ("computed tomography" OR "CT") AND "bone" AND "edema," which were changed by the different databases as necessary. There were no language limitations [9]. One author conducted a gray literature search to identify any further such records, evaluating recent annual meetings of the American Roentgen Ray Society (ARRS) and the Radiological Society of North America (RSNA) and the European Congress of Radiology (ECR). Inclusion criteria-compliant conference abstracts that were not yet accessible in full form were analyzed.

Literatures from each database and other sources were compiled into a list, duplicates were deleted, and the list was initially assessed for relevancy based on the title and abstract. Thereafter, a full-text analysis of possibly relevant papers was performed.

### Inclusion criteria

For papers to be included in this systematic review, the following criteria had to be met: (1) BME was examined around the knee joints; (2) BME was the goal finding and DECT was the index test; (3) MRI served as the gold standard; (4) at least 10 patients were included; and (5) sufficient data could be retrieved to form a 2 × 2 contingency table. Case reports, comments, consensus statements, recommendations, and narrative reviews were rejected as non-original research. If multiple studies presented overlapping data, only the largest or most recent study was included.

### Data extraction

The necessary data were extracted and documented in Excel files that were standardized (version 16.54, Microsoft). The following research features were recorded: first author's surname, publication year and nation, prospective versus retrospective study design, time between DECT and MRI, number of readers, existence of consensus reading, and reader experience. After acute knee injury, patient data including total number and inclusion interval, mean age (Range), number of men and females, and number of knees (regions) with BME were recorded. In addition, true positive (TP), false positive (FP), false negative (FN), true negative (TN), and threshold value were recorded for each evaluation.

### Risk of bias assessment

The methodological quality of the included studies was evaluated using the Quality Assessment of Diagnostic Accuracy Studies (QUADAS)-2 instrument, which is comprised of four distinct domains: (1) patient selection, (2) index test(s), (3) reference standard, and (4) flow and timing [10]. Risk of bias (ROB) was evaluated in each domain, and concerns regarding application were evaluated using signaling questions in the first three domains. These questions were responded with "yes" when there was a low risk of bias/concern, "no" when there was a high risk of bias/concern, or "unclear" when relevant information was not presented in a clear manner. Studies deemed high risk for any signaling question within a domain were deemed to have a high ROB for that domain.

### Statistical analyses

In general, a 2 2 contingency table was created for each study considered. If the study included both qualitative and quantitative analysis, two distinct contingency tables were constructed. Using the bivariate meta-analysis methodology, the pooled sensitivity, specificity, positive likelihood ratio (PLR), negative likelihood ratio (NLR), and diagnostic odds ratio (DOR) were determined (bivariate mixed effects regression model). Moreover, summary receiver operating characteristic (sROC) curves were created, with the area under the curve (AUC) representing the tests' accuracy.

Extensive subgroup analysis was done on the studies to investigate potential sources of variation. And univariate analysis was conducted after the bivariate regression model failed to converge due to the small sample size (< 4) and the absence of values in the 2 × 2 contingency table. In all statistical tests, a two-sided p value 0.05 was deemed statistically significant. Concurrently, a statistical analysis of publication bias was conducted. Stata version 16 (StataCorp, College Station, TX) was utilized to analyze data from the included research, whereas Review Manager Software version 5.4 (Cochrane Collaboration, Oxford, UK) was used to evaluate the methodological quality of the included investigations.

## Results

### Literature search

**Fig. 1:**
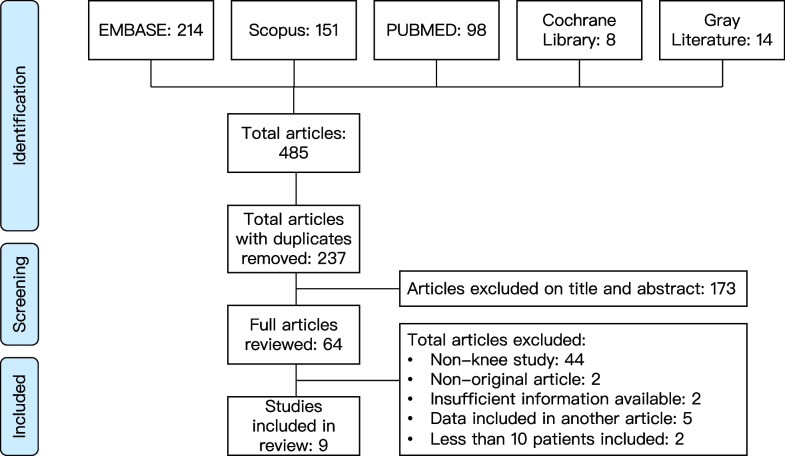
PRISMA flow diagram showing screening and selection of studies included in the analysis

Figure 1 depicts the PRISMA flow diagram for the literature search. By scanning databases and deleting duplicates, a total of 237 articles were uncovered. Following initial screening of titles and abstracts, 64 articles were evaluated further by examining the complete texts using the predetermined criteria, and 9 articles [11–19] were ultimately included for study.

### Study characteristics

Table 1 summarizes the basic features of the included studies. Nine studies with a total of 290 participants with an average age range from 23 to 53 assessed 2,809 bone areas near the knee for BME. There were four studies conducted in European institutions, four studies conducted in Asian institutions, and one research conducted in a North American institution. There were seven prospective studies and two retrospective investigations. The period between DECT and MRI varied each investigation, but was often less than one week. At least two readers participated in each study, with five papers receiving consensus evaluation.

**Table 1:** Patient and study characteristics of included studies

Author, published year	Country	No. patients (inclusion interval)	Mean age (range)	Male/female	No. knees with edema (no. regions)	Study design	Time interval between DECT and MRI	No. readers	Consensus reading	Reader experience (years)
Ai [11]	USA	14 (2009.03–2011.07)	24.6 (18–48)	11/3	NR (36/56 regions)	Retrospective	< 99 days	2	Yes	MSK radiologists (11 + 19)
Bjorkman [12]	Sweden	48 (2017.10–2018.03)	23 (15–37)	26/22	52 (85/192 regions)	Prospective	< 7 days	2	Yes	Resident (3) + Radiologist (> 7)
Booz [13]	Germany	57 (2017.01–2018.05)	50 (20–82)	27/30	36 (197/684 regions)	Retrospective	< 7 days	6	No	Qualitative: 4 residents (3–4) + 2 radiologists (7–10) Quantitative: medical students (2)
Cao [14]	China	32 (2012.04–2012.08)	39.9 (20–60)	24/8	32 (127/384 regions)	Prospective	≤ 6 days	2	Yes	Radiologists (vastly experienced)
Foti [15]	Italy	33 (2019.01–2019.06)	52.2 (31–76)	20/13	25 (85/396 regions)	Prospective	< 6 days	2	Yes	Radiologists (20 + 16)
Juhng [16]	South Korea	23 (NR)	47.4 (14–72)	11/12	NR (84/378 regions)	Prospective	NR	2	No	Radiologists (5 + 25)
Liang [17]	China	23 (2018.07–2019.06)	47 (21–73)	10/13	24 (121/288 regions)	Prospective	NR	5	No	MSK radiologists (8 + 10 + 4 + 5 + 20)
Pache [18]	Germany	21 (2009.01–2009.05)	35.9 (19–60)	16/5	NR (59/236 regions)	Prospective	≤ 5 days	2	No	MSK radiologists (5 + 7)
Wang [19]	China	39 (2018.03–2018.11)	36.3 (13–89)	22/17	NR (43/195 regions)	Prospective	≤ 5 days	3	Yes	Radiologists (> 5)

*No.*—number; *DECT*—dual-energy computed tomography; *MRI*—Magnetic resonance imaging; *NR*—not reported

### Diagnostic performance

**Fig. 2:**
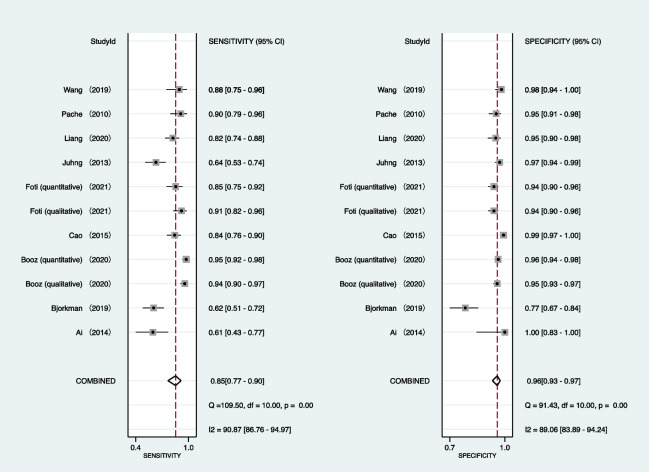
Forest plots of the sensitivity (left) and specificity (right) of dual-energy CT for detecting bone marrow edema in patients with acute knee injury

**Fig. 3:**
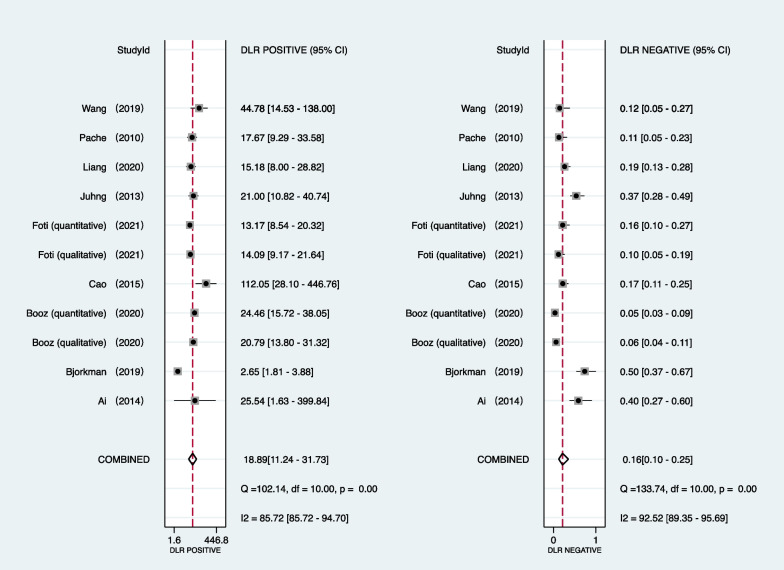
Forest plots of the PLR and NLR of dual-energy CT for detecting bone marrow edema in patients with acute knee injury

**Fig. 4:**
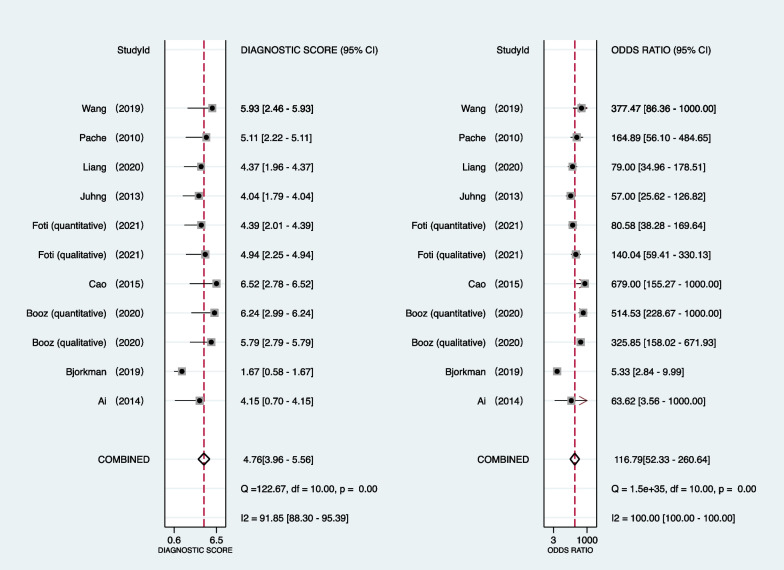
Forest plots of the diagnostic score and DOR of dual-energy CT for detecting bone marrow edema in patients with acute knee injury

**Fig. 5:**
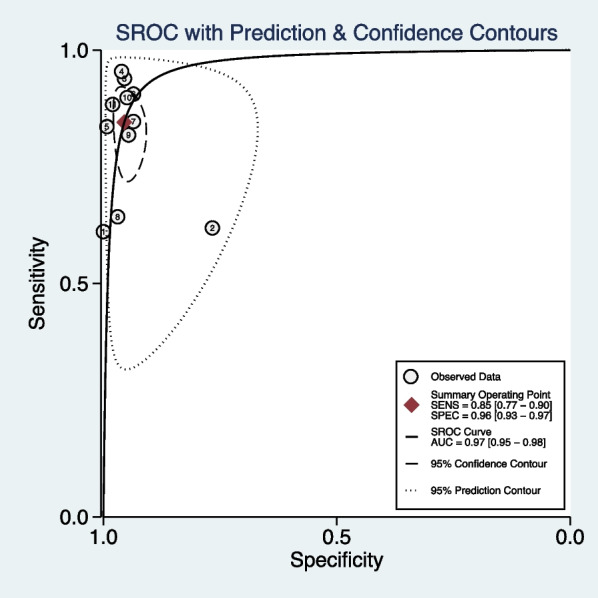
Summarized receiver operating characteristic curve (sROC) of dual-energy CT for detecting bone marrow edema in patients with acute knee injury

Table 2 provides a summary of integrated data for individual investigations by location of pathology. All investigations employed region-based characterization to verify the uniformity of results. As seen in Fig. 2, the pooled sensitivity and specificity of DECT for diagnosing BME in patients with an acute knee injury are 85% (95% confidence interval [CI]: 77–90%) and 96% (95% CI: 93–97%), respectively. In accordance with Figs. 3 and 4, the pooled PLR, NLR, and DOR were 18.89 (95% CI 11.24–31.73), 0.16 (95% CI 0.10–0.25), and 116.79 (95% CI 52.33–260.17), respectively. Figure 5 shows that the AUC for BME detection by DECT in individuals with acute knee injury is 0.97 (95% CI 0.95–0.98). The I^2^ figures for sensitivity and specificity values are 90.87% (95% confidence interval [CI]: 86.76–94.97%, *p* = 0.00) and 89.06% (95% CI: 83.89–94.24%, *p* = 0.00), respectively, showing high heterogeneity among the included studies.

**Table 2:** Integrated data for individual studies by site of pathology

Author	Published year	Site of pathology	No. total regions	No. marrow edema	TP	FP	FN	TN	Threshold value*
Ai	2014	Knee	56	36	22	0	14	20	Qualitative
Bjorkman	2019	Knee–Femur	95	43	26	11	17	41	Qualitative
		Knee–Tibia	96	41	26	14	15	41	Qualitative
Booz	2020	Knee–Femur	342	91	85	11	6	240	Qualitative
		Knee–Tibia	342	106	100	11	6	225	Qualitative
		Knee–Femur	342	91	86	12	5	239	− 42 hu
		Knee–Tibia	342	106	102	7	4	229	− 51 hu
Cao	2015	Knee–Femur	192	49	36	2	13	141	Qualitative
		Knee–Tibia	192	67	61	0	6	125	Qualitative
Foti	2021	Knee	396	85	77	20	8	291	Qualitative
		Knee	396	85	72	20	13	291	− 15 hu
Juhng	2013	Knee–Femur	162	29	17	7	12	126	Qualitative
		Knee–Tibia	162	44	33	3	11	116	Qualitative
		Knee–Femur + Tibia + Patella	378	84	54	9	30	285	
Liang	2020	Knee	288	121	99	9	22	158	Qualitative
Pache	2010	Knee–Femur	114	19	15	2	4	93	− 33 hu
		Knee–Tibia	122	40	38	7	2	75	− 60 HU
Wang	2019	Knee	195	43	38	3	5	149	− 67 HU

*No.*—number; *TP*—true positive; *FP*—false positive; *FN*—false negative; *TN*—true negative; *HU*—Hounsfield unit

*Threshold value for quantitative studies determined as a region of interest measuring a circumference of at least 3 mm and placed at least 1 mm from the cortical margin

The subgroup analysis is shown in Table 3.

**Table 3:** Subgroup analysis of included studies evaluating for causes of presumed variability amongst studies

	Sensitivity (95% CI)	Specificity (95% CI)	AUROC (95% CI)
All	85% (77–90%)	96% (93–97%)	0.97 (0.95–0.98)
Qualitative only	80% (68–88%)	96% (90–98%)	0.95 (0.93–0.97)
Quantitative only	91% (85–95%)	95% (94–97%)	0.98 (0.96–0.99)
Femur only	81% (66–91%)	95% (91–98%)	0.96 (0.94–0.98)
Tibia only	89% (79–95%)	96% (89–98%)	0.97 (0.96–0.99)
North America/Europe	87% (77–93%)	93% (88–95%)	0.96 (0.94–0.97)
Asia	80% (71–87%)	98% (95–99%)	0.97 (0.95–0.98)
Prospective	82% (74–88%)	95% (91–98%)	0.95 (0.92–0.96)
Retrospective*	89% (62–97%)	96% (94–97%)	
Consensus	81% (70–88%)	96% (89–98%)	0.94 (0.91–0.91)
No consensus	88% (77–94%)	96% (95–97%)	0.96 (0.94–0.98)
Mean Age 20–39	79% (67–88%)	96% (88–99%)	0.93 (0.91–0.95)
Mean Age 40–59	88% (79–93%)	95% (94–96%)	0.96 (0.94–0.97)

*Univariate analysis performed when bivariate regression model did not converge due to limited number of studies (< 4) and zero values in the 2 × 2 contingency table

According to each of the six key characteristics, every study was separated into two groups. Due to the small number of research (< 4) within subgroups, retrospective study designs were analyzed using univariate analysis. Studies employing qualitative evaluation methods tended to have a lower sensitivity than quantitative research (80% (68–88%) versus 91% (85–95%), NS), but there was no difference in specificity. There was no difference in sensitivity between studies from North American and European universities and those from Asian institutions (93% (88–95%) versus 98% (95–99%), NS). No other statistically significant differences were detected between the comparison groupings to account for the assumed inter-study variability.

### Risk of bias assessment

**Fig. 6:**
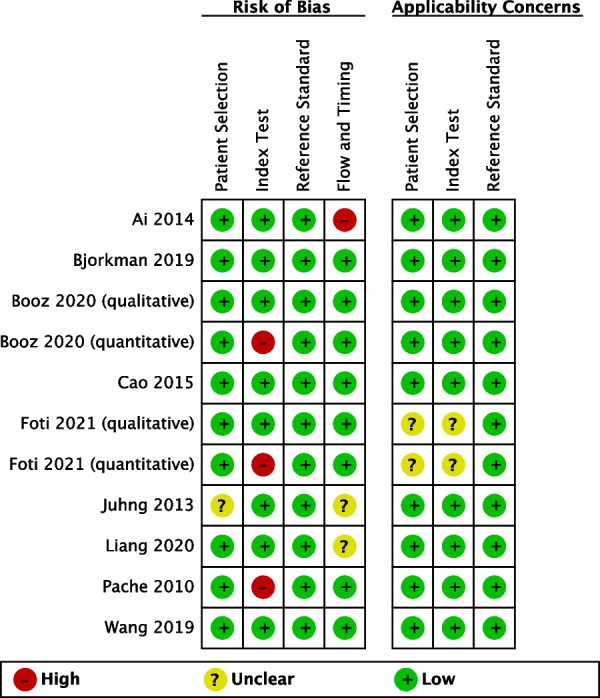
Results of QUADAS-2 tool evaluation of individual studies for risk of bias and applicability. Red in figure indicates high risk, yellow represents unclear risk and green means low risk

Figure 6 displays the outcomes of the QUADAS-2 technique for assessing the risk of bias and applicability of specific research. The majority of studies had a low risk of both bias and applicability. One research was regarded as having a high risk of bias in the flow and timing domains due to an improper time delay between DECT and MRI (more than 2 weeks) [11]. Two studies evaluated both quantitative and qualitative evaluation, but only quantitative evaluation was rated high risk since the threshold is known beforehand [13, 15]. In addition, one more research was deemed to have a high risk of bias for the index test when employing a retrospective criterion [18].

### Publication bias

**Fig. 7:**
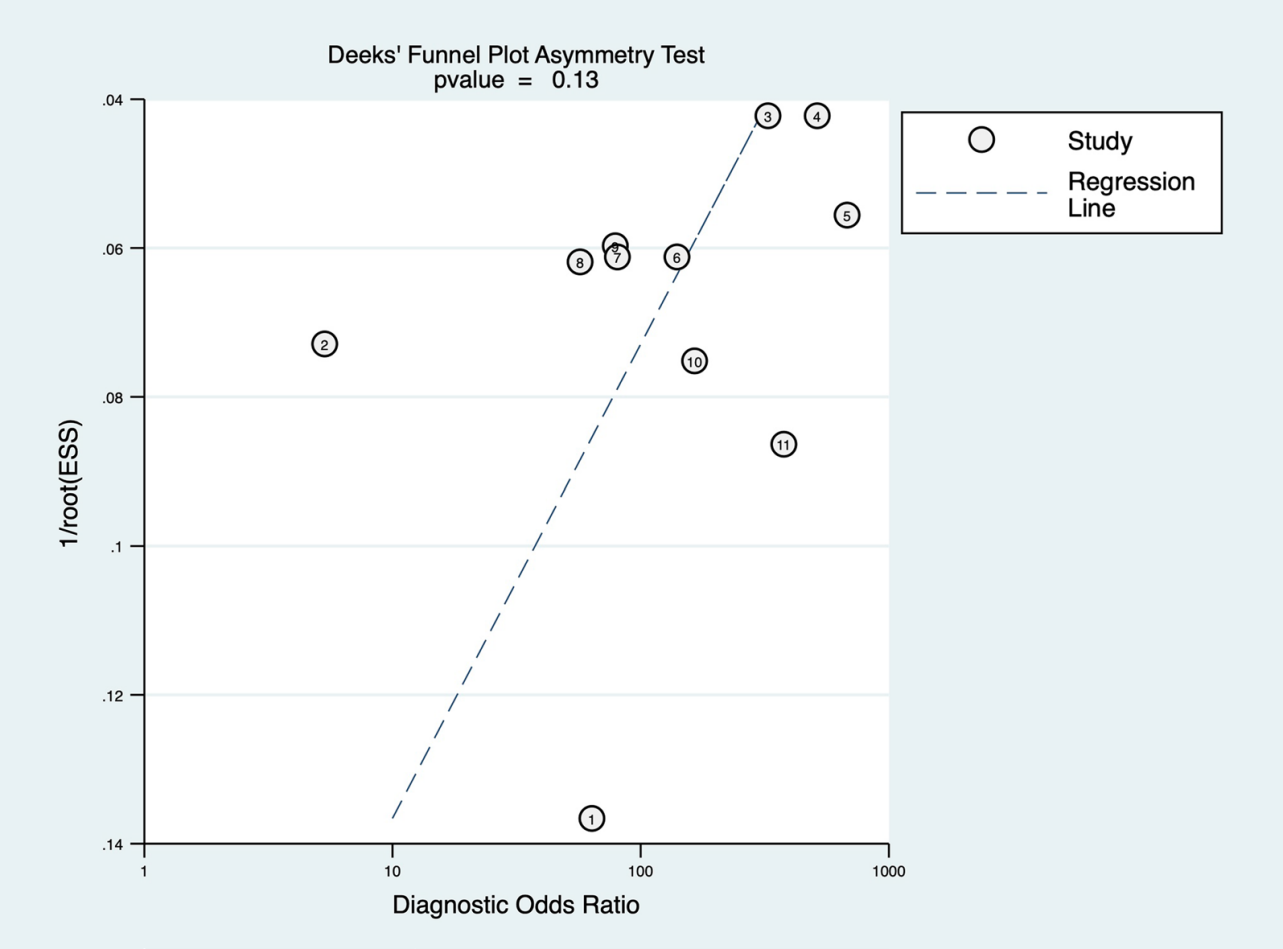
Deek’s funnel plot asymmetry test indicated the existence of publication bias

As shown in Fig. 7, Deek’s funnel plot asymmetry test indicated the existence of publication bias (*p* = 0.13).

## Discussion

Knee injuries are a common health concern for people of all ages, and early diagnosis and treatment can avoid further damage progression. MRI may correctly identify bone marrow edema, a sign of mild bone damage that is globally recognized. However, a lengthy evaluation period, a fixed condition, and the incompatibility of some medical equipment such as pacemakers indicate that there are still limits. Now, DECT is being studied as a potential alternative to MRI using a sophisticated three-material decomposition process that can eliminate elements with photoelectric effects on BME, such as calcium and iodine. The therapeutic utility of DECT for measuring BME in adult patients with an acute knee injury remains debatable, and DECT's accuracy must be demonstrated. Thus, the purpose of this systematic review and meta-analysis was to compile and assess available evidence on the sensitivity of DECT for identifying BME in patients with acute knee injury and to establish whether MRI is still required for these individuals.

This meta-analysis reveals that DECT is extremely specific and reliable for diagnosing BME in patients with acute knee injury when MRI is used as the gold standard, with great pooled specificity (96 (95% CI 93–97%)) and AUC (0.97 (95% CI 0.95–0.98). Our findings suggest that DECT can be utilized as a substitute for MRI in patients with suspected but undetected bone fractures following acute knee injury, particularly when MRI is contraindicated or unavailable. A somewhat decreased sensitivity of DECT for detecting BME when compared to an MRI reference standard (85% (95% CI: 77–90%)) shows that individuals with negative DECT findings but persisting clinical symptoms may still need an MRI to detect more concealed bone damage.

A highlight was the detailed subgroup analysis conducted to investigate potential causes of assumed variability and raise confidence in the generalizability of these results. There were no statistically significant variations between these categories in terms of specificity and sensitivity. Importantly, qualitative research tended to have lower sensitivity than quantitative studies, while North American and European studies tended to have less specificity than Asian studies. This study gives several potential for future research to further assess specific subgroups, including DECT methods not currently involved, and to discover the optimal use and interpretation of DECT in patients with acute knee injury based on patient- and imaging-specific data. In addition, despite the fact that the majority of studies posed a low risk of bias and applicability, a few studies employed retrospective thresholds, and one study used extended intervals between DECT and MRI, which increased the risk of bias in the index test and flow and timing domains, respectively.

In addition, some inherent limitations of DECT maintain MRI as the preferred imaging modality at present, including: (1) the non-specificity of BME for fracture (e.g., bone infarction can result in BME); (2) accompanied bone injury when soft tissues such as meniscal and ligament are frequently injured; (3) increased radiation dose; (4) risk for non-diagnostic virtual non-contrast reconstructions; and (5) limited field of view in virtual non-contrast reconstructions [13, 14, 18]. Under certain conditions, however, our systematic review and meta-analysis demonstrates that DECT can be used as an alternative to MRI for patients with acute knee injury. Individuals with a negative DECT but a high suspicion of bone damage may still need an MRI to assess for concealed fractures.

## Conclusions

Based on the results of this meta-analysis, it is possible to conclude that DECT is accurate for identifying BME in patients with acute knee injury with high sensitivity and specificity, suggesting it may be utilized as an alternative to MRI, especially when MRI is contraindicated or unavailable.

## Data Availability

The datasets used and/or analyzed during the present study are available from the corresponding author on reasonable request.
